# Virtual reality in the management of patients with low back and neck pain: a retrospective analysis of 82 people treated solely in the metaverse

**DOI:** 10.1186/s40945-023-00163-8

**Published:** 2023-05-16

**Authors:** Eran Orr, Tal Arbel, Miki Levy, Yaron Sela, Omer Weissberger, Omer Liran, Jeremy Lewis

**Affiliations:** 1 XRHealth Inc, Boston, MA USA; 2 XRHealth Ltd, Tel Aviv, Israel; 3 School of Psychology, Reichman University, Herzliya, Israel; 4 Cedars-Sinai, Division of Health Services Research, Department of Medicine, Los Angeles, CA USA; 5 Department of Psychiatry and Behavioral Sciences, Cedars-Sinai, Los Angeles, CA USA; 6 Therapy Department, Central London Community Healthcare National Health Service Trust, Finchley Memorial Hospital, London, N12 0JE UK; 7 Clinical Therapies, University of Limerick, Limerick, Ireland

**Keywords:** Virtual reality, Low back pain, Neck pain, Exercise therapy, Musculoskeletal, Physiotherapy and rehabilitation

## Abstract

**Background:**

Clinically, neck pain disorders (NPD) and non-specific low back pain (NS-LBP) are respectively the fourth and first most common conditions associated with the greatest number of years lived with disability. Remote delivery of care may benefit healthcare sustainability, reduce environmental pollution, and free up space for those requiring care non-virtual care.

**Methods:**

A retrospective analysis was performed on 82 participants with NS-LBP and/or NPD who received exercise therapy delivered solely in the metaverse using virtually reality. The study was to determine if this was achievable, safe, had appropriate outcome measures that could be collected, and if there was any early evidence of beneficial effects.

**Results:**

The study demonstrated that virtual reality treatment delivered via the metaverse appears to be safe (no adverse events or side effects). Data for more than 40 outcome measures were collected. Disability from NS-LBP was significantly reduced (Modified Oswestry Low Back Pain Disability Index) by 17.8% (*p* < 0.001) and from NPD (Neck Disability Index) by 23.2% (*p* = 0.02).

**Conclusions:**

The data suggest that this method of providing exercise therapy was feasible, and safe (no adverse events reported), that complete reports were obtained from a large selection of patients, and that software acquired outcomes were obtainable over a range of time points. Further prospective research is necessary to better understand our clinical findings.

**Supplementary Information:**

The online version contains supplementary material available at 10.1186/s40945-023-00163-8.



## Background

Neck pain disorders (NPD) are a serious public health problem that were ranked fourth (out of 301 health conditions) across 188 countries in the global, regional, and national incidence, prevalence, and years lived with disability project [1]. Of comparable concern are data demonstrating that the burden of NPD has not decreased since 1990 [1]. Occurrence throughout a lifetime ranges from 14.2% to 71% [2], and for the majority of the neck disorders there is an absence of an identifiable underlying disease or abnormal anatomical structure. Consequently, NPD are classified as ‘non-specific’[3].

NPD may also be classified by mechanism of onset, pathoanatomy, duration, mechanism, clinical prediction rules, and subgroups [4]. NPD may also be classified as neck pain with mobility deficits, movement co-ordination impairments, headaches, and radicular pain [5].

As it is often impossible to identify the cause(s) of neck pain it should be considered as a symptom and not a disease. The pain in NPD is most commonly experienced between the occipital region and upper back. The distribution of pain may be unilateral, bilateral (symmetrical or asymmetrical) and may present with or without arm pain. The experience of pain may be short lived, recurring, and in some cases, persistent. Management most commonly involves advice, reassurance, pharmacological intervention, and exercise (with or without manual therapy) [4]. Exercise, whether specific or general, is effective in reducing pain and disability, and improving function in NPD [4, 6]. Currently there is no consensus on the most beneficial exercise regimen [7].

Low back pain (LBP) most commonly presents as pain between the lower costal margin and the gluteal folds and may be associated with or without leg pain. LBP is the leading cause of disability worldwide. Estimates indicate that 80 percent of the population will experience LBP at some stage of their lives. LBP is classified multifariously, commonly categorized by duration and presentation. An episode of acute LBP lasts up to six weeks, subacute, from six to 12 weeks, and persistent (also known as chronic) reserved for symptoms lasting longer than 12 weeks. For the majority, the outcome is favorable, however, approximately 32% develop persistent LBP [8]. Risk factors for the transition to persistent LBP include obesity, smoking, LBP with leg pain, high baseline disability, anxiety and depression, and discordant care that does not align with guidelines [8]. Examples of discordant care include prescription of opioids, benzodiazepines, systemic corticosteroid medications, diagnostic imaging, radiofrequency denervation, spinal fusion surgery, and specialist referral [8, 9].

LBP is also classified by presentation, with one in 20 people being diagnosed with nerve root related back pain (sciatica) and one in 100, diagnosed with serious spinal pathology. The majority of people (85%) are categorized as having non-specific low back pain (NS-LBP).

NS-LBP is defined as low back pain not attributable to a recognizable, known specific pathology (e.g., infection, tumor, osteoporosis, fracture, or inflammatory disorder).

NS-LBP should also be thought of as a symptom and not as a disease, as myriad factors may be associated with the onset and perpetuation of symptoms. Intervention focuses on education, reassurance, pharmacological management, and when required non-surgical management. Surgery is rarely required, and the excessive use of imaging, opioids, and invasive procedures is a global problem [10, 11]. Based on published guidelines, first stage concordant care for most people with NS-LBP includes education and advice, to remain as active as possible, and avoid bed rest [9, 12]. Of concern, despite the global effort and the plethora of published research to better understand the causes of LBP and concomitant initiatives to provide consistently effective management, LBP disability rates as with NPD disability, have not decreased in over 30 years [1]. Multiple reasons for the poor translation from guidelines to practice have been identified, with authors suggesting that shifting resources from unnecessary care to guideline-concordant care would have widespread positive benefit [9].

In the United States of America, healthcare expenditure for LBP and NPD is ranked the third highest (after diabetes and ischemic heart disease) with USD $87.6 billion (uncertainty index $67.5 billion to $94.1 billion) spent in 2013 [13]. Spending on LBP and NPD increased exponentially from 1996 to 2013, coming second only to the increase in spending on diabetes [13]. The majority of healthcare spending for LBP and NPD (60.5%) was on outpatient (ambulatory) care [13].

Common to all guidelines for LBP and NPD is the advice to remain active [5, 9] and exercising in a virtual environment is a potential and novel method to achieve this objective.

Immersive virtual reality (VR) places an individual in a simulated environment, achieved through wearing a head mounted display (HMD). Within the environment virtual hands interact with the environment and manipulate virtual objects. Such environments have been used for education [14], pain relief [15], and reducing fear [16]. Due to its increasing accessibility, affordability, and ease of use, VR is gradually being introduced into musculoskeletal practice for both assessment and management, and to facilitate physical activity treatment [17–19].

The metaverse is the name given to a parallel digital world where an individual’s avatar can interact with other individual’s avatars. It has been described as a three-dimensional version of the Internet with leaders in this technology (Google™, Meta™, Microsoft™, and Apple™) suggesting that in the future people will enter this digital world to do nearly everything done in the real world. Whether this vision is realized or not will be a question answered in the future. However, human services such as healthcare, once only available in brick-and-mortar structures are becoming increasingly available virtually in the metaverse [20].

Telemedicine involves the use of digital communication to deliver healthcare services remotely and is considered a key area in which the potential of the metaverse is indisputable [21]. Turolla et al. [22] and Certitelli et al. [23] have described the advantages, challenges, and future of telerehabilitation. Although many uncertainties and disadvantages are considered, advantages may include reduction of hospitalization rates and readmissions, early discharge, immediate access to rehabilitation services, education, improved progression monitoring, and providing feedback.

Clearly, to reduce the disability and exponentially increasing costs associated with NS-LBP and NPD, new strategies are needed. Virtual treatment in the metaverse may contribute to this need in a myriad of ways. To gain insight into its potential role, the aim of this study was to generate early real-world data from the health records of people seeking care for low back and neck pain, who received VR treatments conducted in the metaverse.

The primary aim of this study was to answer the following question:

- Would participants seeking care for low back and neck pain be willing to participate in virtual reality treatment delivered entirely in the metaverse?

The secondary aims were to:

- Determine if there were any side effects, adverse events, or serious adverse events.
- Learn if outcome measures could be applied and completed remotely.
- Gain formative information if virtual reality treatment delivered in the metaverse had a positive benefit.

To achieve these aims, data were analyzed retrospectively from health records (also known as a medical records review) generated in three countries for participants experiencing LBP and NPD.

## Methods

A retrospective health and medical records review was conducted with a waiver of consent on participants who had remote VR rehabilitation services between July 2020 and May 2022 at XRHealth clinics (https://www.xr.health/), an international hybrid technology and healthcare company specializing in the provision of healthcare through the metaverse. In this unique healthcare delivery model, licensed clinicians augment remote healthcare interventions with VR applications, after screening for contraindications for use of the technology. This report follows STROBE checklist guidelines [24].

### Setting and participants

Participants in this retrospective analysis of health records were from three countries: the United States of America, Israel, and Australia. Their data were included if they were receiving treatment for low back and or neck pain. This may have been for acute, sub-acute, or persistent symptoms. The data did not permit a further analysis of these characteristics. Each participant reported a primary reason for seeking treatment, which was recorded. If there were other health concerns these were also documented under secondary concerns.

Inclusion criteria to be included in this analysis were participants seeking care for low back pain, and / or neck pain, as a primary concern.

### Outcome measures

The disability outcome measurements listed in Table 2 were completed digitally by the patients and the results entered into the patient’s health records. The impairment outcome measurements listed in Table 3 were recorded when patients were in the virtual environments from movements obtained automatically from the head mounted display and hand controllers. The reliability and validity of these measurements remains a focus of current research.

The main outcome measures for this retrospective analysis were:

#### Modified Oswestry Low Back Pain Disability Index (MOLBPDI)

This consists of 10 patient-completed questions in which the response options are presented as 6-point Likert scales. Scores range from 0% (no disability) to 100% (most severe disability). The MOLBPDI is designed for use in people experiencing acute and persistent low back pain. It has been suggested that a threshold of 50% improvement in MOLBPDI may be a valid measure for defining a successful outcome for patients with LBP [25]. The MOLBPDI was available in the language of choice for each participant [26].

#### Neck Disability Index (NDI)

The NDI has 10 items and patients rate their pain from 0 (no pain) to 5 (worst imaginable pain). Individual item responses are summed to a total score, where 0 points indicate no limitation, and 50 points indicate complete disability. The NPI has been designed for use in acute and persistent neck pain and for those diagnosed with cervical radiculopathy. The threshold for minimally important clinical differences is reported to be 5.5 [27]. The NPI was available in the language of choice for each participant [28].

A secondary aim of the analysis was to review the health records to determine if there were any side effects, adverse events, or serious adverse events associated with the treatment package. A side effect was defined as an undesired but known response to VR treatment that may occur in some people when using VR. Examples include anxiety and emotional distress, dizziness, headaches, eye strain, nausea, sweating, pallor, loss of balance. Participants are advised of known side effects and their frequency, if that data is available. Side effects may be mild, moderate, or serious / severe. An adverse event was defined as an undesired and unpredicted response to VR treatment that may occur in some people when using VR. Examples include any unwanted event with an unknown risk that did not result in hospitalization, permanent disability, or death. A serious adverse event was defined as an event that resulted in hospitalization, permanent disability, or death. Over time and when more data are available, adverse events may be recategorized as side effects.

Other outcome measures were administered based on the presenting condition of the patient (Supplemental Table S1). Outcome measures were collected at baseline, reassessed every 30 days, and upon discharge from care.

### Virtual reality hardware and therapeutic software

Participants were provided with the Pico Neo 2 (ByteDance) head mounted display (HMD) and hand controllers. Treatment software was uploaded to the HMD. Supplemental Table S2 provides a description of the XRHealth therapeutic software provided for the participants. The software is registered with the FDA (U.S. Food and Drug Administration), AMAR (Ministry of Health, Medical devices Department, Israel), and has an ARTG (Australian Register of Therapeutic Goods) Certificate.

### Application of treatment

Based on the assessment process, the clinician (licensed physical therapists/physiotherapists and occupational therapists) would determine which software package(s) to prescribe for the participants. Clinicians utilized training applications (apps) that were relevant for the required rehabilitation. This therefore may have included one or more apps. For example, an individual diagnosed with stress and anxiety may be prescribed relaxation software such as Mindset, while an individual presenting with symptoms in the cervical region including pain may be prescribed Rotate together with Luna. Moreover, each application allows the clinician to adjust specific training parameters, such as training area, speed, and cognitive challenge, according to the specific healthcare needs of the patient.

**Fig. 1:**
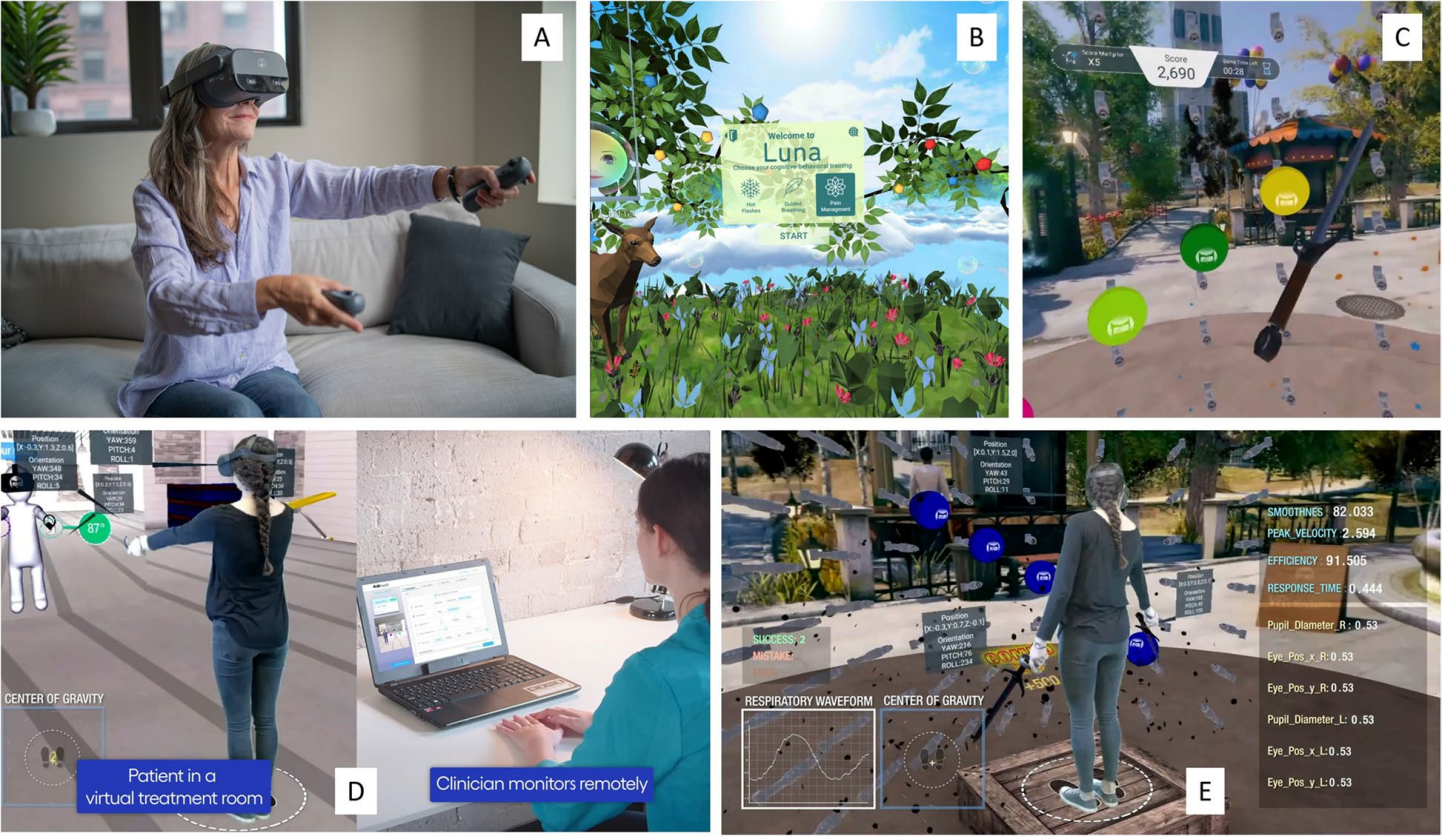
depicts selected images of the hardware and software used by participants with low back and neck pain in this retrospective analysis of healthcare records. Legend: **A** Patient wearing head mounted display and holding hand controllers interacting with VR. **B** Image of the ‘Luna’ software. **C** Image of the ‘Balloon Blast’ software. **D** Image of patient in a VR treatment room and clinician performing remote monitoring. **E** Data collection in VR

The clinicians providing the VR management were employees of XRHealth. Each had gone through online self-directed theoretical and practical training programs. In addition to this training, they underwent patient simulations and real patient supervised training. They are only permitted to use the hardware and software independently once the training is successfully completed (Fig. 1).

#### Data sources

Data were derived from a manual search and data extraction of the participant’s anonymized healthcare records.

#### Study size

This was a retrospective review of healthcare records, and it was unknown before the records were searched how many records would meet the inclusion criteria.

#### Statistical methods

Data were analysed using R software (V. 4.1.0) and R studio software (V. 2022.02.3).

Descriptive statistics were reported using means (M) and standard deviations (SD) for continuous variables and count (N) and percentage (%) for categorical variables. A per-protocol approach was used, analysing patients who completed the treatment course and had sufficient data points both at the beginning and end of the treatment.

Outliers that exceeded 3 standard deviations were examined using box plots and excluded. To examine the effect of the VR intervention on patient reported outcomes, VR clinical measurements and functional outcomes, paired t-tests between the baseline (pre) and the final (post) measurements were conducted. For each comparison, the mean differences were calculated between measurements using percentages, points and 95% confidence intervals for mean difference.

*P*-values that were lower than 5% were considered significant. *P*-values lower than 10% were reported as a marginally significant.

## Results

### Participants

Data was gathered on participants with low back and neck pain who underwent treatment (provided through virtual reality in the metaverse) and who presented to clinics in the United States, Australia, and Israel between July 2020 and May 2022. The health records of all participants who had presented for treatment for NS-LBP and NPD were included. The sample consisted of 82 participants, with a mean age of 55.8 years (SD = 14.4 years). The cohort consisted of 66.7% (*n* = 55) females, 28.4% (*n* = 23) males and 4.9% (*n* = 4) who self-identified as ‘other’.

Overall, the participants underwent a mean of 127.1 days treatment (SD = 68.3), with a mean of 22.7 telehealth appointments with their clinician (SD = 12.6) and participated in a mean of 186.2 virtual reality sessions.

Data was extracted on the number of sessions in which participants received treatment with a clinician present (in the metaverse) and the number without. For example, the mean number of virtual sessions with a clinician using the ‘Light Punch’ software was 5.9 (SD = 7.9) and without a clinician was 9.1 (SD = 20.3). Table 1 details demographic and clinical baseline characteristics of the participants (*n* = 82).

**Table 1:** Demographic and clinical baseline characteristics of the participants

	***N* = 82**
**Age (years), mean ± SD**	55.8 ± 14.4
**Gender (self-selected), n (%)**	
Female	54 (66.7)
Male	23 (28.4)
Non-binary/other	4 (4.9)
**Days in treatment, mean ± SD**	127.1 ± 68.3
**Total appointments, mean ± SD**	22.7 ± 12.6
**Total number of treatments in the metaverse, mean ± SD**	186.2 ± 213.4
**Number of virtual sessions with a clinician for each app used, mean ± SD**	
Light Punch	5.9 ± 7.9
Luna	1.2 ± 1.9
Color Match	14.8 ± 19.7
Memorize	5.5 ± 10.2
Balloon Blast	24.7 ± 20.4
Rotate	11.2 ± 12.7
**Number of virtual sessions in the metaverse without a clinician for each app used, mean ± SD**	
Light Punch	9.0 ± 20.3
Luna	5.3 ± 11.5
Color Match	23.9 ± 44.5
Memorize	12.1 ± 35.4
Mindset	6.7 ± 9.7
Balloon Blast	35.8 ± 51.8
Rotate	20.3 ± 40.7
Reducept	6.3 ± 20.8

*SD* standard deviation, *app* application

## Main results including descriptive and outcome data

### Effect on patient reported outcomes

Table 2 presents pre-post intervention change for the Patient Reported Outcomes.

**Table 2:** Pre-post intervention change of patient reported outcomes

Outcome	N	Pre M (SD)	Post M (SD)	Mean Difference	Delta (95% CI)	t	*P*-Value
Modified Oswestry Low Back Pain Disability Index	50	22.5 (8.9)	18.5 (9.8)	17.8	4.0 (2.4, 5.7)	4.8	< 0.001
NIH PROMIS CAT – Pain Interference	22	1.4 (0.7)	1.1 (0.7)	17.9	0.2 (-0.1, 0.6)	1.5	0.15
NIH PROMIS CAT – Fatigue	17	1.3 (0.7)	1.2 (0.7)	5.6	0.1 (-0.3, 0.04)	0.4	0.71
Brief Pain Inventory – Pain Interference	14	7.3 (7.2)	9.5 (12.9)	30.8	-2.3 (-10.0, 5.5)	-0.6	0.54
Brief Pain Inventory – Pain Severity	12	4.9 (1.6)	44 (2.2)	10.1	0.5 (-0.5, -1.5)	1.1	0.30
PROMIS CAT v1.0—Sleep Disturbance	14	1.2 (0.7)	0.9 (0.5)	20.2	0.3 (-0.2, -0.7)	1.2	0.25
NIH PROMIS – Pain Intensity	12	0.7 (0.4)	0.5 (0.4)	28.9	0.2 (-0.1, -0.5)	1.4	0.18
PROMIS CAT v1.0—Anxiety	11	1.1 (0.7)	1.1 (0.6)	5.7	-.01 (-0.4, -0.3)	-0.3	0.75
Neck Disability Index	12	20.6 (7.7)	15.8 (7.1)	23.3	4.8 (0.9, 8.8)	2.1	0.02
Lower Extremity Functional Scale (LEFS)	6	38.2 (23.1)	48.8 (22.8)	27.8	-10.7 (-27.5, 6.1)	-1.6	0.16
PROMIS v1.0 Sleep disturbance-Short Form (4a)	4	0.98 (0.71)	0.9 (0.6)	10.2	0.1 (-0.5, 0.7)	0.5	0.64

Mean difference is calculated by post score minus pre score

*NIH* National Institute of Health, *PROMIS* Patient-Reported Outcomes Measurement Information System, *CAT* computer adaptive test

The trend in nine (out of 11) outcome measures was improvement over the course of the VR treatment, and in two cases the reported improvement reached statistical significance.

A significant improvement was found for the mean Modified Oswestry Low Back Pain Disability Index, with a lower score (indicating improvement) recorded after the treatment (pre-post delta of 17.8%, *p* < 0.001). The second significant improvement was found at the Neck Disability Index, with a mean lower score (improvement) following treatment, in comparison with the score before treatment (pre-post delta of 23.3%, *p* = 0.02).

### Effect on impairment measurements

Table 3 details the pre-post intervention effect on impairment outcome measurements. Pre-treatment and post-treatment scores were calculated as the average of the first and last three data points, respectively.

**Table 3:** Effect on VR clinical measurements

Pain Test	*N*	Pre M (SD)	Post M (SD)	Mean Difference	Delta (95% CI)	t	*p*
**Pain**							
VAS (start of session)	68	4.5 (2.1)	3.8 (1.9)	15.1	0.7 (0.3, 1.04)	3.7	< 0.001
VAS (end of session)	66	4.1 (1.7)	3.6 (1.9)	11.5	0.5 (0.1, 0.8)	2.8	0.007
Delta VAS (In session)	67	0.20 (0.7)	-0.02 (0.5)	110.0	0.2 (0.03, 0.4)	2.3	0.02
**Range of Movement**							
Flexion left	11	164.0 (24.4)	166.0 (17.5)	1.2	-1.6 (-21.9, 18.7)	-0.2	0.87
Flexion right	11	164.0 (25.0)	167.0 (17.7)	1.8	-2.7 (-23.4, 18.1)	-0.3	0.78
Abduction left	10	183.0 (24.4)	187.0 (32.5)	2.2	-4.1 (-29.8, 21.6)	-0.4	0.73
Abduction right	11	174.0 (37.1)	192.0 (29.6)	10.3	-17.9 (-47.2, 11.3)	-1.4	0.20
Horizontal abduction left	10	75.1 (32.8)	85.6 (16.3)	13.9	-10.4 (-31.3, 10.4)	-1.1	0.29
Horizontal abduction right	9	81.6 (29.0)	92.2 (29.0)	12.9	-10.5 (-39.5, 18.4)	-0.8	0.43
**Quality of movement**							
Left hand	80	72.4 (12.0)	72.3 (11.3)	0.1	0.2 (-2.5, 2.9)	0.1	0.91
Right hand	80	72.4 (12.4)	73.4 (10.7)	1.4	-1.0 (-3.9, 1.9)	-0.7	0.49
Average	81	70.8 (11.0)	72.7 (10.4)	2.7	-1.9 (-4.6, 0.9)	-1.4	0.18
Peak velocity left hand	78	1.8 (0.6)	1.9 (0.6)	5.6	-0.1 (-0.2, 0.04)	-1.4	0.16
Peak velocity right hand	78	1.9 (0.7)	1.9 (0.6)	3.2	-0.1 (-0.2, 0.1)	-0.8	0.41
Peak velocity average	78	1.8 (0.6)	1.9 (0.6)	2.7	-0.01 (-0.2, 0.1)	-0.7	0.49
Response time left hand	80	621.0 (179.0)	489.0 (143.0)	21.3	131.9 (87.8, 176.1)	5.6	< 0.001
Response time right hand	79	603.0 (169.0)	475.0 (130.0)	21.2	127.7 (89.2, 166.3)	6.6	< 0.001
Response time average	81	513.0 (229.0)	393.0 (181.0)	23.4	120.3 (67.1, 173.5)	4.5	< 0.001
Efficiency left hand	74	80.9 (9.5)	80.9 (7.5)	0	0.01 (-1.9, 1.9)	.006	0.99
Efficiency right hand	75	81.3 (8.9)	81.8 (6.4)	-0.5	-0.5 (-2.5, 1.5)	-0.5	0.63
Efficiency average	75	80.9 (8.95)	81.1 (6.9)	0.6	-0.1 (-2.1, 1.8)	-0.1	0.90
Action time left hand	62	1050.0 (256.0)	1005.0 (218.0)	0.3	44.8 (-13.9, 103.6)	1.5	0.13
Action time right hand	61	1036.0 (256.0)	993.0 (205.0)	4.3	43.3 (-16.9, 103.4)	1.4	0.16
Action time average	73	744.0 (520.0)	726.0 (443.0)	4.2	17.4 (-86.9, 121.8)	0.3	0.74
Speed	62	11.6 (4.98)	12.7 (4.7)	2.4	-1.1 (-2.8, 0.50)	-1.4	0.17
**Neck rotation**							
ROM rotation right	17	64.4 (9.42)	70.6 (8.9)	9.6	9.6 (-10.4, -1.8)	-3.0	0.007
ROM rotation left	16	65.1 (8.75)	70.5 (10.9)	8.3	8.3 (-10.6, -0.1)	-2.2	0.04
ROM extension	17	50.3 (11.4)	54.9 (14.3)	9.2	9.2 (-10.7, 1.5)	1.6	0.13
ROM flexion	17	55.6 (9.30)	64.5 (10.1)	16.0	16.0 (-12.7, -5.1)	-4.9	< 0.001
ROM side bending right	15	32.4 (11.0)	34.9 (15.2)	7.7	7.7 (-8.2, 3.2)	-0.9	0.37
ROM side bending left	15	32.5 (12.7)	34.8 (16.4)	7.1	7.1 (-8.8, 4.2)	-0.8	0.46
Session accuracy	43	88.8 (7.4)	92.0 (5.9)	3.6	3.6 (-5.4, -1.1)	-3.0	0.004
Session constant error	39	2.6 (1.3)	1.9 (0.6)	26.3	26.3 (0.3, 1.1)	3.5	0.001
Final speed level	69	7.5 (4.1)	9.3 (4.8)	23.9	23.9 (-3.1, -0.5)	-2.8	0.007
**Memory**							
Final number of items	35	3.2 (1.3)	4.3 (1.8)	33.7	33.7 (-1.6, -0.5)	-3.9	< 0.001

Mean difference is calculated by post score minus pre score

*CI* confidence interval, *ROM* range of motion, *VAS* visual analogue score

The findings suggest that, overall participants did not report any deterioration over the course of treatment using VR in the metaverse. In 13 (of 35) impairment outcome measurements, significant improvements were recorded, indicating lower pain, faster response time of arms movements, and better range of motion for neck rotation, session accuracy, speed, and total number of items to memorize.

For most of the remaining outcome measures, although statistical significance was not achieved, the trend was of improving health and function.

### Effect on functional outcome measurements

Table 4 details pre-post intervention change on functional outcome measurements.

**Table 4:** Effect on functional outcome measurements

Outcome	N	Pre M (SD)	Post M (SD)	Pre-Post Improvement (%)	Mean Difference (95% CI)	t	*P*-Value
**General**							
Sit to Stand	31	9.6 (3.8)	11.8 (3.7)	22.8	-2.2 (-3.5, -0.9)	-3.5	0.002
Rapid cognition	2	9.0 (1.4)	9.5 (0.7)	5.6	-0.5 (-6.9, 5.6)	-1.0	0.5
**Right hand**							
Hand to Head	7	22.4 (7.9)	25.7 (12.1)	14.7	-3.3 (-19.3, 12.7)	-0.5	0.6
SLS	7	22.5 (22.4)	24.4 (21.3)	8.4	-1.9 (-5.8, 2.0)	-1.2	0.3
HBB	3	15.3 (13.1)	22.3 (10.0)	45.8	-7.0 (-37.1, 23.1)	-1.0	0.4
**Left hand**							
Hand to Head	7	21.4 (6.7)	23.4 (10.9)	9.4	-2.0 (-11.8, 7.8)	-0.5	0.6
SLS	8	15.7 (19.6)	18.5 (16.9)	17.8	-2.8 (-10.3, 4.6)	-0.9	0.4
HBB	3	16.3 (13.8)	25.0 (11.4)	53.4	-8.7 (-48.3, 30.9)	-0.9	0.5

Mean difference is calculated by post score minus pre score

*CI* confidence interval, *HBB* hand behind back, *SLS* single leg stand

The findings of this analysis of the medical records suggest that participants did not report any deterioration over the course of treatment using VR in the metaverse.

A significant improvement was identified for the sit-to-stand test after intervention (pre-post delta of 22.8%, *p* < 0.001). The remaining outcome measures, although not statistically significant, indicated a trend of improving health and function.

### Side effects, adverse events, or serious adverse events associated with the treatment

This review of health records for 82 patients treated with VR in the metaverse did not identify side effects, adverse events, or serious adverse events associated with the treatment.

## Discussion

Musculoskeletal LBP is associated with more years lived with disability than any other health condition, and NPD are considered to be the fourth [3]. Despite the international equivalent of many tens of millions of US dollars having been awarded for spinal research, the levels of disability have not decreased between 1990 and 2017 [29, 30].

Although the level of evidence is considered to be low to moderate, and associated with a high risk of bias, for many people with NS-LBP and NPD, exercise therapy appears to be an effective treatment [6, 31]. There does not appear to be a preference for one exercise type over another [31, 32]. There is emerging evidence that supervised, and to a lesser extent, unsupervised exercise therapy, combined with frequent communication, delivered via telehealth, is associated with better outcomes than a control population [33].

These data suggest that although not a panacea, exercise therapy is safe, as well as practical, and is associated with other health benefits. It is also a relatively low-cost intervention, which would contribute to healthcare sustainability [10, 11]. As no exercise program has demonstrated definitive superiority and an increasing amount of care for these conditions is being delivered remotely [34], investigating the role of VR delivered remotely in the metaverse, may ensure more efficient use of health resources, than providing care in traditional brick and mortar establishments.

### Key results

The primary aim of this study was to review the health records of individuals seeking care for NS-LBP and NPD to determine their willingness to receive treatment facilitated by VR delivered entirely in the metaverse. Early data suggested that participants were as engaged in treatment that included virtual sessions with clinicians as those that were self managed. Most of the sessions were conducted independently without input from a clinician. With a mean age of 55.8 years (SD = 14.4) the majority of the participants would be classified as ‘middle aged’. Their engagement with non-supervised sessions suggests that the technology was accessible and understood by those participating.

A secondary aim of the analysis was to review the health records to determine if there were any side effects, adverse events, or serious adverse events associated with the treatment package. The review of the health records for this patient group did not identify any documentation reporting side effects, adverse, or serious adverse events. Due to the nature of the available data, a conclusion cannot be made with confidence if the results indicate that there were no such effects or events, or they occurred but were not communicated to the clinicians or were reported and not recorded by clinicians. This will need to be a focus of future research.

The data suggest that no deterioration over the course of treatment was reported by the participants, and although many of the outcome measures did not achieve statistical significance, the trend over time was one of improving health. Again, the results are not clear if this was related to improvement as a result of the interventions or was a natural improvement over time, or a combination of the two.

Another secondary aim was to learn if outcome measures could be applied and completed remotely. This was an important consideration as outcome measures provide one of the clearest methods of monitoring the history of a condition and determining the effect of intervention. The analysis of the data suggested that participants were fully engaged and provided outcome measure data at multiple time points. The review suggested that there was very little missing data across multiple outcome measures and multiple timepoints. The VR system and software used in the management of NS-LBP and NPD has the advantage of collecting substantial quantities of impairment data (range of motion, speed of movement, peak velocity, response time, movement efficiency), which reduces the burden on both participants and clinicians. These findings are important as the breadth and depth of data generated by participants and by the software will be essential when data is collected and analyzed prospectively.

The data suggest that the soft- and hardware can record cervical range of motion data in multiple planes and speeds of movement. The reliability of these measurements was not tested but should become a feature of future research as Rondoni et al. [35] have concluded both expensive (> €500) and inexpensive (< €500) systems demonstrate comparable intra- and inter tester reliability. Using their classification system, the system used in the current study would be considered inexpensive and knowledge of its reliability would be useful clinically.

The final aim was to gain inceptive information on outcomes when treatment was provided through virtual reality delivered in the metaverse. It was encouraging that the participants reported significant reductions in both the Modified Oswestry Low Back Pain Disability Index, and the Neck Disability Index following the VR exercise treatment they received via the metaverse. There were significant improvements in pain levels, as there were with some, but not all, ranges of movement, quality of movement, and limb response times. Research has reported that a single VR session was effective in increasing cervical range of motion in people without symptoms. Further research is needed to determine the potential of VR in the assessment and management of people with neck pain [36].

Another finding of interest was the significant improvement in memory with an increase in the number of items remembered. There was also a significant improvement for the sit-to-stand test after intervention (pre-post delta of 22.8%, *p* < 0.001). The remaining outcome measures, although not statistically significant, indicated a trend of improving health and function. These findings may be related to treatment differences conducted in traditional environments compared with virtual environments. VR may induce an external focus (directed at the movement effect), unlike traditional therapy that focuses on promoting an internal focus (directed at the performer’s body movements). Motor learning studies suggest that such an external focus is more effective as it facilitates automaticity, which in this case would be the movement being performed involuntarily that becomes unconscious, innate, and ingrained [37].

From a Bayesian perspective, our brains generate expectations, which are probabilistic predications about the body [38, 39]. Examples of negative predictions include “*Turning my neck always leads to neck pain,”* and *“Bending forwards always exacerbates my back pain.”* One aim of clinical processes such as symptom modification procedures [40] is to overcome the patient’s expectations of pain and symptoms during movement. Virtual reality has the potential to disrupt negative probabilistic predictions associated with symptomatic movement and change the external attention focus [41] for some people experiencing NS-LBP and NPC. This hypothesis requires testing in future research.

No approach has yet demonstrated definitive reduction in the disability associated with NS-LBP and NPC and as such the findings from this retrospective analysis of health records are encouraging. These findings will be more fully understood following investigations using more robust research methods. Furthermore, the results presented in this analysis come from patients treated in high income countries with relatively easy access to Internet services and the required hardware and software, than in lower income countries. If this technology proves to be an effective method of providing education and management for these common musculoskeletal conditions, the barriers to fair and equitable provision of healthcare within and between countries needs to be overcome.

### Limitations

The retrospective analyses of data are subject to substantial limitations, which include issues relating to blinding, selection bias, respondent bias, recall bias, and response bias. Another limitation is that because the study design did not include a non-treatment comparison, outcomes may be explainable by natural improvement over time or contextual effects such as placebo. There were potential confounding variables that were unmeasured and uncontrolled in the analysis. These include unequal populations in the three countries where participants received treatments. Other examples include age of participants, duration of symptoms, concurrent comorbidities, and potential lack of standardization of condition education within and between clinicians for the various conditions being treated.

Another important consideration is that although the findings suggest that statistical significance was achieved in a number of patient-reported outcomes, it is uncertain if clinical changes occurred during the VR intervention. Although this was not the aim of this study, it must be addressed in future more robust prospective clinical trials.

## Conclusions

A retrospective analysis was conducted on the health records of 82 participants presenting with NS-LBP and NPD. The data suggest that participants were willing to participate and fully engaged with the rehabilitation protocols that were provided entirely in the metaverse using VR equipment and dedicated VR software. Participants were able to understand how to apply, use and progress independently with the majority of treatments being provided in the absence of any clinical input. A substantial amount of clinical data was collected which was provided by participants across multiple timepoints, using multiple outcome measures. Extensive data generated by the software was collected, which has the advantage of reducing both clinician and participant burden. The findings suggest that there were no side effects, adverse, or serious adverse events, and some of the participants reported and software generated outcomes were associated with significant improvement. Most notably in this population, the interventions were associated with significant improvements for the Modified Oswestry Low Back Pain Disability Index, and the Neck Disability Index. This suggests that VR should be considered in the management of NS-LBP and NPD, though further prospective research (multiple baseline case studies, cohort and randomized clinical trials) is necessary to better understand these clinical findings.

## Supplementary Information

**Additional file 1: Supplemental Table S1.** Outcome Measures Used in the Retrospective Analysis. Supplemental Table S2. XRHealth Therapeutic Software Applications Used by the Participants.

## Data Availability

The datasets generated during and/or analyzed during the current study are not publicly available but are available from the corresponding author on reasonable request.

## Abbreviations

AMAR: Ministry of Health, Medical devices Department, Israel
Apps: Applications
ARTG: Australian Register of Therapeutic Goods
FDA: United States Food and Drug Administration
HMD: Head mounted display
LBP: Lower back pain
M: Mean
N: Count
NPD: Neck pain disorders
NS-LBP: Non-specific low back pain
SD: Standard deviation
US: United States
VR: Virtual reality
