# Supplementary material for: Virtual reality in the management of patients with low back and neck pain: a retrospective analysis of 82 people treated solely in the metaverse
Source: Arch Physiother. 2023 May 16;13:11. doi: 10.1186/s40945-023-00163-8 (PMC10189988; doi:10.1186/s40945-023-00163-8)
Supplement: Supplementary file 1 — Additional file 1: Supplemental Table S1. Outcome Measures Used in the Retrospective Analysis. Supplemental Table S2. XRHealth Therapeutic Software Applications Used by the Participants. [file 40945_2023_163_MOESM1_ESM.docx]

**Supplementary Materials**

**Virtual reality in the management of patients with low back and neck pain: A retrospective analysis of 82 people treated solely in the metaverse**

**Table of Contents**

1. Supplemental Table S1: Outcome Measures Used in the Retrospective Analysis
2. Supplemental Table S2. XRHealth Therapeutic Software Applications Used by the Participants
3. Supplemental References

**1. Supplemental Table S1: Outcome measures used in the retrospective analysis**

| **Outcome Measure** | **Description** |
| --- | --- |
| Brief Pain Inventory – Pain Severity^1^ | BPI allows patients to rate the severity of their pain and the degree to which their pain interferes with common dimensions of feeling and function. |
| Lower Extremity Functional Scale (LEFS)^2^ | Used to assess the functional impairment in individuals with musculoskeletal lower limb dysfunction. |
| Modified Oswestry Low Back Pain Disability Index^3^ | Provides a subjective percentage score of level of function (disability) in activities of daily living in those rehabilitating from low back pain |
| Neck Disability Index^4^ | A 10-item questionnaire that measures a patient's self-reported neck pain related disability. |
| NIH PROMIS – Pain Intensity^5^ | PROMIS^®^ is a publicly available system for assessment of patient-reported health status for physical, mental, and social well-being. |
| NIH PROMIS CAT – Pain Interference^5^ |  |
| PROMIS CAT v1.0 - Sleep Disturbance^5^ |  |
| Brief Pain Inventory – Pain Interference^5^ |  |
| NIH PROMIS CAT – Fatigue^5^ |  |
| PROMIS CAT v1.0 - Anxiety^5^ |  |
| PROMIS v1.0 Sleep disturbance-SF (4a)^5^ |  |

NIH, National Institute of Health; PROMIS, Patient-Reported Outcomes Measurement Information System; CAT, computer adaptive test

**2. Supplemental Table S2. XRHealth Therapeutic Software Applications Used by the Participants**

| **Therapeutic Software**  **Company Code and Name** | **Description** |
| --- | --- |
| CT-610* (ReAct) | Provides sensory motor cognitive exercises for the upper limbs and whole body in a virtual environment |
| CB-510 (Luna) | Contributes to the reduction in pain through distraction and exercises performed in a virtual environment |
| MC-320* (Color Match) | Provides sensory motor cognitive exercises for the whole body in a virtual environment |
| CT-620 (Memorize) | Provides cognitive exercises in a virtual environment |
| PD-810 (Mindset) | Assists in relaxation and management of pain through distraction, relaxation, and meditation exercises |
| MT-220* (Balloon Blast) | Provides sensory motor cognitive exercises for the whole body in a virtual environment |
| N-140* (Rotate) | Provides sensory motor cognitive exercises for the cervical region in a virtual environment |
| MD-710 (Relax8) | Assists in relaxation and management of pain and physical discomfort through distraction and supporting meditation and relaxation exercises. |

All the motion software (*) generates motion tracking and movement kinematics data. These data were not analyzed in this retrospective analysis but will be used to inform the design of future case control, cohort, and randomized clinical trials.

**3. Supplementary References**

1. Cleeland CS, Ryan K. The brief pain inventory. *Pain Research Group*. 1991;20:143-147.
2. Binkley JM, Stratford PW, Lott SA, Riddle DL, Network NAORR. The Lower Extremity Functional Scale (LEFS): scale development, measurement properties, and clinical application. Physical therapy. 1999;79:371-383.
3. Fairbank J, Couper J, Davies JB, O’Brien JP. The Oswestry low back pain disability questionnaire. *Physiotherapy*. 1980;66:271-273.
4. Vernon H, Mior S. The Neck Disability Index: a study of reliability and validity. Journal of manipulative and physiological therapeutics. 1991;
5. Cella D, Riley W, Stone A, et al. The Patient-Reported Outcomes Measurement Information System (PROMIS) developed and tested its first wave of adult self-reported health outcome item banks: 2005–2008. Journal of clinical epidemiology. 2010;63:1179-1194
